# Blunt traumatic isolated duodenal perforation treated by multimodal endoscopic approach

**DOI:** 10.1055/a-2068-8123

**Published:** 2023-04-26

**Authors:** Durante Donnarumma, Omar Ksissa, Lorenzo Dioscoridi, Edoardo Forti, Osvaldo Chiara, Luigi Raparelli, Massimiliano Mutignani

**Affiliations:** 1 Digestive Endoscopy Unit, ASST Grande Ospedale Metropolitano Niguarda, Milan, Italy; 2 Trauma Center and Emergency Surgery, ASST Grande Ospedale Metropolitano Niguarda, Milan, Italy; 3 Department of General Surgery and Oncology, Ospedale San Camillo de Lellis, Rieti, Italy

Duodenal trauma usually consists of retroperitoneal lesions, which represent a small percentage of abdominal injuries. These injuries are difficult to manage by surgery, so endoscopy can be an useful alternative 1 2 3 .

A 17-year-old man presented to the Emergency Department of a first-level care hospital after a collision with a teammate during a football match. As a result of a gradual onset of sepsis during in-hospital observation, he underwent an abdominal computed tomography (CT) scan that showed post-traumatic duodenal perforation. He therefore underwent emergency laparotomy, with two duodenal lesions found and treated by direct drainage (using transduodenal transabdominal catheters), and additional surgical drains were placed ( Fig. 1 a ). Given our expertise in the management of enteral perforations 4 and to avoid re-do surgery, an endoscopic approach was then proposed and the patient was referred to our third-level care hospital.

**Fig. 1:**
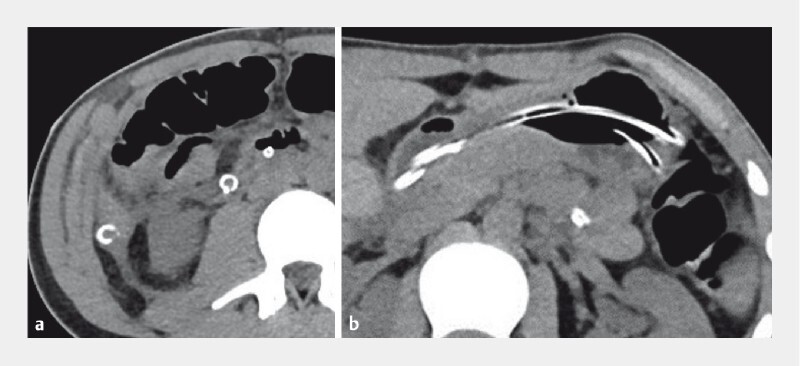
Computed tomography scan images showing: **a** the position of the two transabdominal transduodenal Foley catheters with the distal ends located one in the descending portion and one in the ascending portion of the duodenum; **b** a large residual fluid collection extending from the horizontal portion of the duodenum to the right abdominal quadrants.

An additional abdominal CT scan confirmed the correct position of the two catheters, along with a large residual fluid collection ( Fig. 1 b ). Firstly, frontal view endoscopy, using 3.8-mm operative gastroscope, confirmed the two duodenal perforations, with the catheters passing through these having been fixed by surgical stitches. The two catheters were endoscopically removed after 0.035-inch guidewires had been placed through them, with one of them replaced by a tubular drain that extended 4–5 cm from the duodenal lesion.

Closure was firstly attempted by over-the-scope (OTS) clipping. The first OTS clip (OTSC system, Ovesco) was released at the level of the most proximal lesion; however, it was not possible to place a second OTS clip at the level of the second perforation because the distal part of the first clip was too close to the edge of the second perforation. The endoscopic strategy was therefore changed to stenting. A 4.2-mm duodenoscope was used, but cannulation of the major papilla was possible only on the pancreatic side. After septotomy had been performed, a 7-Fr, 20-cm pancreatic stent was placed with its distal end in the gastric cavity. To overcome the lack of a biliary drain, a two-way 18-Fr nasoduodenal tube was positioned near the site of the duodenal perforations and mild suction (−50 mmHg) was maintained to dry the bile. A 22-mm, 8-cm fully covered metal enteral stent (Niti-S enteral stent; Euromedical) was placed straddling the perforations. A second 18-Fr two-way nasoduodenal tube was left in the lumen of the enteral stent to maintain continuous suction (−125 mmHg) and to let the duodenal walls adhere to the stent itself ( Video 1 ) 5 .

**Video 1** Positions of the stents (metal duodenal stent and plastic pancreatic stent) and drainage tubes (nasoduodenal tube near the perforations and two-way nasoduodenal tube in the lumen of the enteral stent) used to endoscopically manage post-traumatic duodenal perforation.


Monitoring of the drain outputs showed no output, even on the first postprocedural day. The surgical drains were gradually removed, with only the new paraduodenal drain left in place by the 16th post-procedural day. On that day, a further abdominal CT scan showed significant reduction of the intra-abdominal collections, with no sign of any residual leaks after the administration of contrast medium ( Fig. 2 ). Both tubes and stents were therefore removed. The OTS clip still appeared to be in place. The patient, having commenced eating per os on the day of the procedure, was discharged in good overall condition after 17 days of hospitalization.

**Fig. 2:**
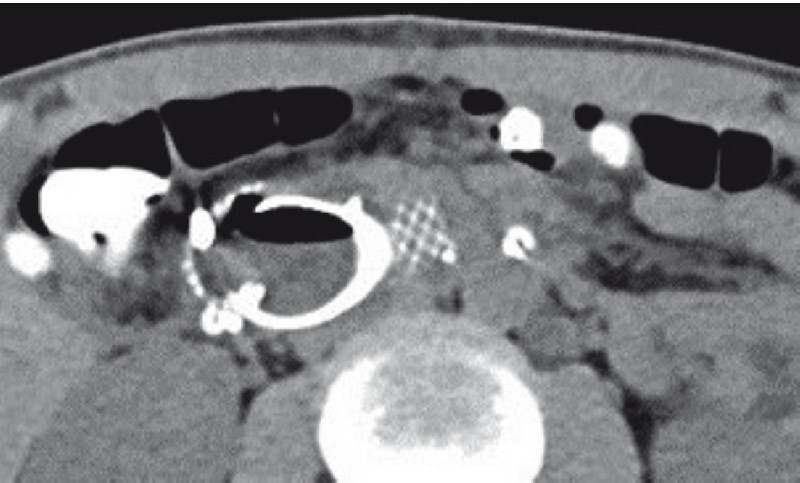
Follow-up computed tomography scan 15 days after the procedure showing no retroperitoneal or intraperitoneal fluid collections, with the self-expandable metal duodenal stent remaining in position.

This case shows how a multimodal endoscopic approach can be used for traumatic duodenal injury, with good clinical results achieved in a relatively short time.

Endoscopy_UCTN_Code_CPL_1AH_2AG

## Notes

Endoscopy E-Videos

https://eref.thieme.de/e-videos
*E-Videos* is an open access online section of the journal *Endoscopy* , reporting on interesting cases and new techniques in gastroenterological endoscopy. All papers include a high-quality video and are published with a Creative Commons CC-BY license. Endoscopy E-Videos qualify for HINARI discounts and waivers and eligibility is automatically checked during the submission process. We grant 100% waivers to articles whose corresponding authors are based in Group A countries and 50% waivers to those who are based in Group B countries as classified by Research4Life (see: https://www.research4life.org/access/eligibility/ ). This section has its own submission website at https://mc.manuscriptcentral.com/e-videos
